# Analysis of the Effect of Backpack Design with Reduced Load Moment Arm on Spinal Alignment

**DOI:** 10.3390/ijerph16224351

**Published:** 2019-11-07

**Authors:** Kyung-hun Kim, Jihyeon Ann, Sang-hun Jang

**Affiliations:** 1 Department of Physical Therapy, Gimcheon university, 214, Daehak-ro, Gimcheon 39528, Korea; huni040@naver.com; 2 Department of Biomedical Science and Research Institute for Bioscience and Biotechnology, Hallym University, Chuncheon, Gangwon 24252, Korea; jh-ahn@hallym.ac.kr

**Keywords:** moment arm, lumbar, alignment, backpack

## Abstract

In this study, we designed a backpack that can reduce the moment arm of backpack load by placing the center of gravity of the backpack close to the axis of the spine. In order to investigate the effect of sagittal spinal alignment compared with the general backpack, we conducted the study using radiological images. The participants in this study were 18 adults (8 males and 10 females). The subjects participated in the experiment without carrying the backpack, wearing the normal backpack, and wearing a backpack designed to reduce the load moment arm by placing the center of gravity close to the body. Spinal alignment parameters were measured and analyzed using 3D radiography measurement software based on radiographic images taken under three conditions. The overall angle of lumbar lordosis, upper arc, lower arc, difference between pelvic incidence and lumbar lordosis, lower cervical lordosis, and sagittal vertical axis were measured. In the case of wearing the backpack rather than without the backpack, there was a significant difference in the overall angle of lumbar lordosis, lower arc, lower cervical spine angle, difference between pelvic incidence and lumbar lordosis, and sagittal vertical axis. In the case of wearing the backpack with reduced moment arm, the overall angle and lower arc of lumbar lordosis were significantly increased compared to those with the normal backpack. The difference between pelvic incidence and lumbar lordosis was significantly decreased. The results showed that a normal backpack caused imbalance of sagittal spinal alignment, and the backpack reducing the load moment arm by placing the backpack’s center of gravity close to the vertebral joint played a positive role in reducing the change of lumbar alignment compared with the normal backpack.

## 1. Introduction

Due to the recent increase of sedentary life, the normal alignment of the spine is damaged, and this abnormal posture causes pain and musculoskeletal problems [1]. Recently, many studies have been conducted on spinal alignment in the sagittal plane as well as deformation in the frontal plane, such as scoliosis [2]. In particular, much research has been conducted on the importance of lumbar lordosis [3].

In general, lumbar lordosis is defined as the angle between L1–S1, and thoracic kyphosis is defined as the angle between T4–T12 [4]. However, some studies have reported that measuring lumbar lordosis using inflection points is more effective in assessing spinal alignment in patients than in measuring traditional lumbar lordosis [5]. It is also important to use spino-pelvic parameters to understand the alignment of the sagittal plane of the lumbar spine. Pelvic incidence is the angle between the vertical line at the midpoint of the sacral plate and the line connecting the same point to the center of the bicoxofemoral axis. Pelvic incidence increases little by little during growth but hardly changes when growth is complete. Another spino-pelvic parameter often used with pelvic incidence is the sacral slope. The sacral slope changes with the posture and position of the pelvis. Pelvic incidence represents the pelvic morphology, and sacral slope represents the pelvic orientation. Pelvic incidence, sacral slope, and lumbar lordosis are closely related to each other [2,5]. The condition of the lumbar spine is also clinically important because it is known to have an important effect on cervical spine alignment associated with forehead posture, which has become more frequent due to increased computer use [6].

The alignment of the spine is greatly affected by backpacks, which are an important means of carrying. In a previous study, hyperlordotic spinal posture increased significantly after walking with wearing a backpack [7]. This hyperlordotic curvature of the lumbar spine causes tension on the anterior side of the spinal column and compression on the posterior surface. If this posture is continued, discomfort in the musculoskeletal system may be caused and the risk of injury may be increased because of increased pressure on the zygopophysial joints and increased stress on the posterior edge of the joint [8]. There was also a study investigating the effect of wearing a backpack on cervical spine alignment. After wearing the backpack, the change in the head-on-neck angle was reported to be insignificant if there was sufficient trunk angle change for balance [8]. Contrary to this, some studies showed that head-on-neck angles tend to increase and lumbar lordosis tends to decrease while wearing a backpack [9,10].

Thus, discussions about the alignment of the lumbar and cervical spine with wearing backpacks are ongoing; however, the conclusions vary. The above study used an anatomic maker of the skin rather than an accurate measurement method such as radiation; however, previous studies have reported that using anatomic markers without images like radiation was quite limited in analyzing spinal alignment [11]. Therefore, accurate spine alignment measurement using radiographic images is necessary.

It is recommended that the weight of the backpack not exceed 10–15% of user’s weight [12]. Excessive backpack weight is known to adversely affect the trunk and neck, or spinal alignment [8], leading to back pain or other musculoskeletal injury [13]. Like this, the weight of the contents of the backpack are well established; however, the research on the design of the backpack is insufficient. One of the most important elements of backpack design is load placement [8]. The position of the backpack’s load and the distance from the spine’s axis are very important factors in determining the moment arm and torque of the vertebral joint. Therefore, if we devise a design that keeps the backpack’s load position close to the spine’s axis, it can be expected to have a positive effect on reducing the load on the spine and preventing deformation of the spine alignment.

In this study, the backpack was designed to reduce the moment arm of the backpack load by keeping the backpack load position close to the axis of the spine. In order to investigate how it affects spinal alignment compared with general backpacks, we conducted a study using radiological images.

## 2. Materials and Methods

### 2.1. Subjects

Eighteen adults (8 males and 10 females) over 19 years old participated in this study. Subjects who had severe communication disorders such as cognitive impairment or aphasia, congenital malformations or diseases of the spine, pelvis, feet, orthopedic or neurosurgery diseases of the spine and legs, severe limitations in the range of joint movement, and who were judged to be inappropriate to participate in this study by the researcher were excluded from this study. This study was conducted with the approval of the Bioethics Committee of Gimcheon University (GU-201709-HRa-06-02). All subjects were fully informed about the experiment and agreed to participate in the study.

### 2.2. Protocol

**Figure 1:**
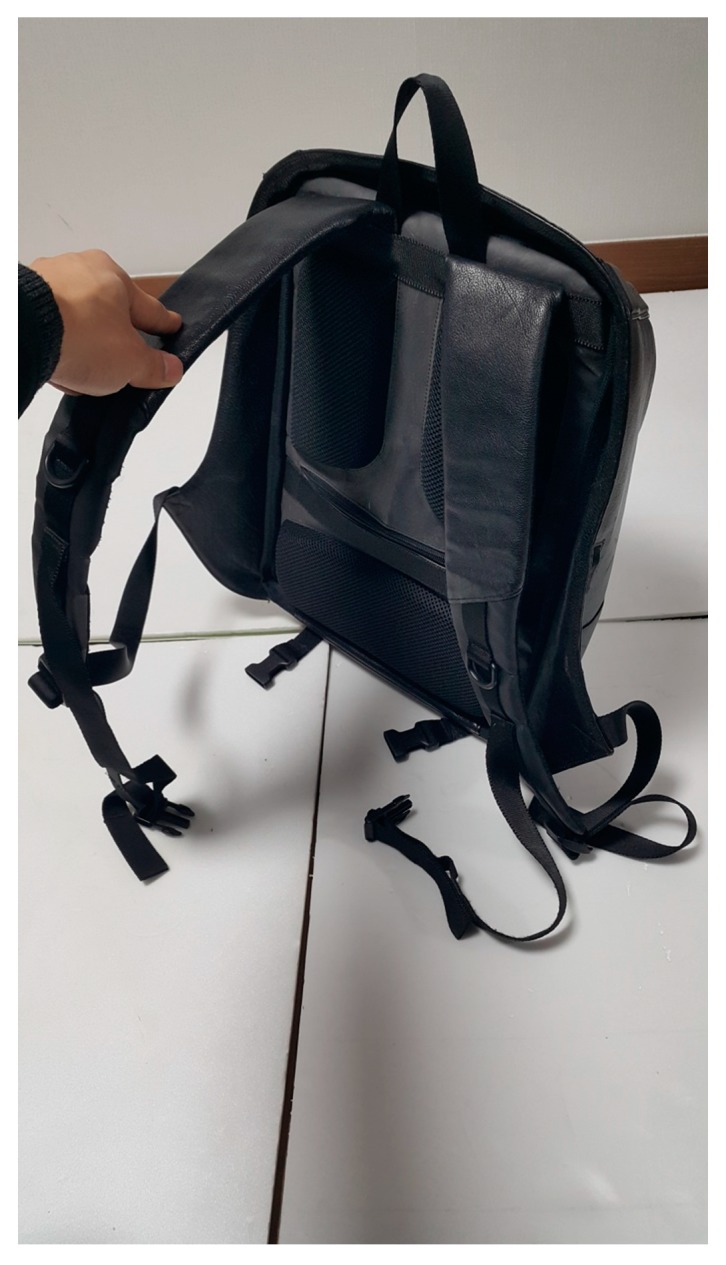
Backpack of typical design.

**Figure 2:**
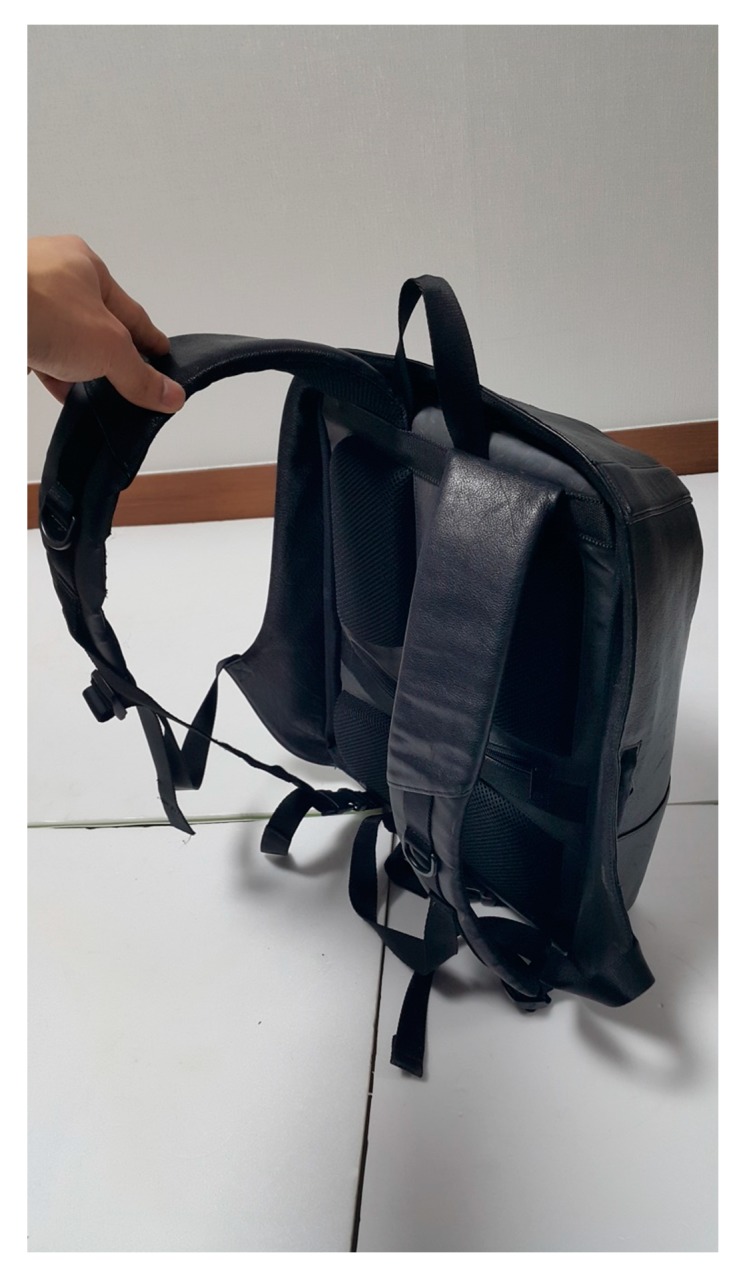
Backpack designed to reduce the moment arm.

The subjects participated in the experiment without carrying the backpack, wearing the normal backpack, and wearing a backpack designed to reduce the load moment arm by placing the center of gravity close to the body. Typical backpacks are 30 cm width, 15 cm depth, and 45 cm height, with the design having a strap starting from the top of the backpack connecting to the lower part on the side (Figure 1). The backpack in the study was designed to reduce the moment arm by placing the center of gravity close to the body, having the same specification as a typical backpack. The strap starting at the top of the backpack is split into two straps in the middle, with one strap attached to the bottom of the side and the other strap to the side of the bottom (Figure 2). It was designed to reduce the load on the vertebral joint and reduce the load moment arm by minimizing the separation between the backpack and the torso.

In a previous study on the weight of a backpack, the weight of the backpack was mainly studied with weights of 5%, 10%, and 15% of the weight of the subject. Usually over 10% has been shown to affect the subject’s spine [14]. Therefore, in this study, the total weight of the backpack was determined to be 10% of the subject’s weight. In order to control the weight of the bag at 10% of the weight, an acrylic plate (28 × 28 × 1 cm, 980 g), a human body equivalent material, was used in the bag. To control the weight, the number of acrylic plates was increased and decreased. The acrylic phantom, a large area of human body equivalent material, was used to prevent the overall weight of the bag from being biased.

Radiographic images were acquired in all 3 conditions: without carrying the backpack, with the normal backpack, and with the backpack with reduced moment arms. Digital X-ray radiography (ysio-max, Siemens, Erlangen, German) was used to acquire the lateral projection radiograph of the whole spine of the subject. As a test method, the centerline of the X-ray was positioned between thoracic spine no. 7–10 according to the subject’s height, and the two images of X-ray irradiation in both up/down directions were stitched to express the whole image of the spine. To minimize the distortion of the image caused by the diverging phenomenon of the X-ray, the focus to detector distance (FID) was set to 229 cm, and the external auricular meatus and femur head in the X-ray field size were used as reference points to include the whole spine of the subject. In this study, both feet of the subject stood at shoulder width so that the weight of the bag was evenly distributed throughout the spine, and the subject was instructed to keep straightening their knee for weighting baring. In order to minimize the overlap of the shoulder and humorous parts with the thoracic spine column in the image, the position of the subject’s hand was placed on the support located at the height of the shoulder, forming an angle of about 30 degrees between the humorous long axis and the coronal plane.

### 2.3. Measurement

**Figure 3:**
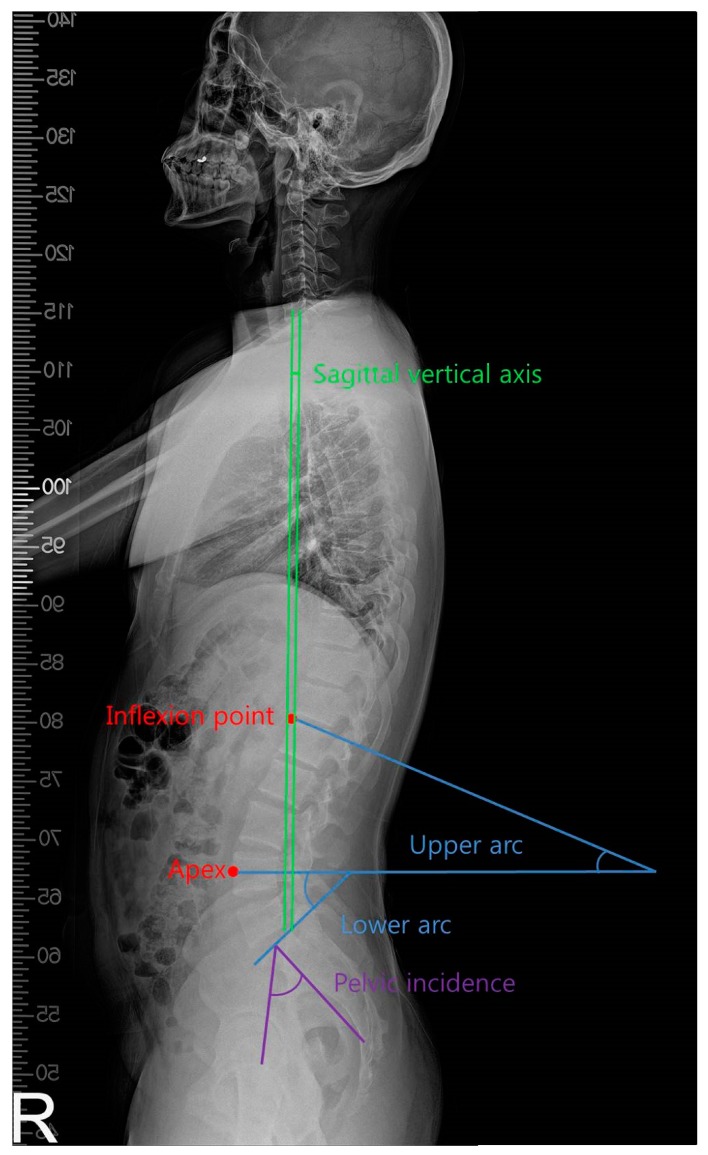
Measurement of the sagittal spinal alignment.

Spinal alignment indices were measured and analyzed using 3D radiography measurement software (Xelis, Infinity, Seoul, Korea) based on radiographic images taken under 3 conditions: without carrying the backpack, with the normal backpack, and with the backpack with reduced moment arms. An expert with more than two years of radiologist experience marked the apex of the lumbar lordosis and the inflection point on the sagittal image (i.e., the point from lordosis to kyphosis). The upper arc of lumbar lordosis is the angle formed by the horizontal line in the apex of lumbar lordosis and parallel to the upper edge of the lower spine at the inflection point. The lower arc is the angle formed by the horizontal line in the apex of lumbar lordosis and parallel to the upper edge of the first sacrum. The overall lumbar lordosis was presented by adding the lower and upper arcs. Pelvic incidence is the angle between the vertical line at the midpoint of the sacral plate and the line connecting the same point to the center of the bicoxofemoral axis [2]. The difference between pelvic incidence and lumbar lordosis is important in the sagittal alignment of the spine. The larger the difference, the more the sagittal imbalance occurs [15]. In addition, to determine the alignment of the cervical spine, we measured the angles formed by the lower cervical lordosis, that is, two tangent lines drawn from the C2 posterior corner and the C7 posterior corner [4]. This method is widely used to measure forward head posture [16,17]. In addition, to determine the sagittal balance of the entire spine, we measured the sagittal vertical axis, i.e., the shortest distance between the vertical line from the center of the 7th cervical vertebra and the posterior upper edge of the sacral spine. The normal range was defined as 5 cm forward and 2 cm backward [18] (Figure 3).

### 2.4. Statistical Methods

Statistical analysis of all collected data was performed using the SPSS version 20.0 statistics program for Windows (IBM Corp., Armork, NY, USA). The general characteristics of the subjects were presented as mean and standard deviation of all variables measured using descriptive statistics. Repeated measure ANOVA was used to compare each spinal alignment under the three conditions. In order to test the statistical significance, the significance level α was set to .05 for all analyses.

## 3. Results

### 3.1. General Characteristics of Subjects

The subjects of the study were 18 adults including 10 females and 8 males. The subjects were 22.8 ± 1.8 years old, 165.9 ± 8.2 cm in height and 66.7 ± 12.2 kg in weight. The subjects’ weights ranged from 48 kg to 85 kg. The weight of the backpack was adjusted to 10% of the weight by increasing or decreasing the number of acrylic plates.

### 3.2. Comparison of Cervical Spine Alignment

Radiographic images were analyzed to compare the lower cervical spine alignment under three conditions: the absence of a backpack and with backpacks of two different designs. The lower cervical spine angle was significantly decreased with the backpack compared to without the backpack. There was no significant difference in cervical spine alignment by backpack design (Table 1).

### 3.3. Comparison of Lumbar Spine Alignment

Lumbar lordosis was reduced with a normal backpack compared with no backpack. However, the overall lumbar lordosis was significantly increased when wearing the backpack with reduced moment arm compared with when wearing the normal backpack. The same pattern appeared in the lower lumbar spine, but the change of the total lumbar spine was affected by the lower lumbar spine. There was no significant difference in the upper lumbar spine (Table 2).

### 3.4. Comparison of Differences between Pelvic Incidence and Lumbar Lordosis

The difference between pelvic incidence and lumbar lordosis was significantly increased with a normal backpack compared with no backpack. However, when wearing a backpack with reduced moment arms, there was no significant difference compared to the state without the backpack (Table 3).

### 3.5. Comparison of Sagittal Vertical Axis

In the state of wearing the backpack, the shortest distance between the sagittal vertical axis, i.e., the centerline of the 7th cervical vertebra, and the posterior upper corner of the sacral spine increased significantly compared to the state of no backpack. However, there was no significant difference in sagittal vertical axis by backpack design (Table 4).

## 4. Discussion

A backpack is important equipment that can contain educational materials, sports equipment, and personal items [19]. A typical two strap backpack is worn on the back of the body and the center of gravity is moved to the back of the base of support due to the weight of the backpack [14]. The posterior load has a significant association with postural changes and sagittal alignment of the spine. Previous studies have reported that backpack weight is a very important factor in inducing dorsal pain and lower back pain, which could potentially lead to tissue damage [19]. Therefore, it is clinically very important to wear a backpack and check the posture change, or spinal alignment. The external load applied to the spine plays an important role as the load moment arm associated with the load center, as well as the weight of the load itself [20,21]. In this study, we designed a backpack with reduced load moment arms by pulling the load center forward to investigate the effect on spinal alignment compared with conventional backpacks.

Previous studies reported that increasing thoracic kyphosis, lumbar lordosis and scoliosis did not contribute significantly to the increase of lower back pain or dorsal pain. However, there is a limitation in the previous studies in that the alignment and clinical features of the spine were confirmed using anatomic markers on the surface of the back rather than the radiological method on sagittal and frontal curvature [10,19]. Therefore, in order to solve the limitations of previous studies, this study attempted to confirm the accurate spine alignment using radiation.

The sagittal balance of the spine is very important for spinal health. It has been reported in previous studies that patients with spinal sagittal imbalance should restore lumbar lordosis in order to correct sagittal imbalance [3]. In previous studies, thoracic kyphosis was measured as between T4‒T12 and lumbar lordosis was measured as between L1–S1 [22]. Recently, the bones that make up the lumbar lordosis were reported to vary from 1 to 8 instead of just 5 in the lumbar spine [5]. These data suggest that analyzing spinal alignment by generalizing only after T1 to T12 and only after L1 to L5 is too simple for proper understanding of spinal alignment. Another method is to use the inflexion point [23]. In previous studies, reliability of the sagittal alignment of the vertebrae and pelvis using this method has been reported to have adequate interobserver and intraobsever reliability as interobserver reliability was between 0.92 and 0.99 and the intraobsever reliability was between 0.93 and 0.99 [2]. In this study, the lumbar curve was measured using the inflection point method.

In a previous study, wearing a backpack reduced lumbar lordosisLBL by flexing lumbar to move the back load into the base of supportBOS [9,10]. Some studies have also reported that backpack loads represented torque increasing lordosis, but thoracic kyphosis increased to maintain balance, and this change in thoracic caused flexion lever-arm effects and contributed to the reduction of lumbar lordosis [11]. In this study, only lumbar lordosis was decreased after wearing the backpack, which is consistent with the results of the previous study. As mentioned earlier, a decrease in lumbar lordosis caused spinal sagittal imbalance. Long-term use of the backpack may have a negative effect on spinal alignment. In this study, lumbar lordosis was measured by dividing it into upper and lower arcs. Lumbar lordosis is enlarged toward the distal part, and it is known that the two segmental angles of the 4–5th lumbar spine and the first lumbar spine and the first sacral spine account for about two-thirds of the total lumbar lordosis [24]. In particular, the sacral angle is the most important part of the overall lumbar lordosis [5]. In this study, when the general backpack was worn, both the upper and lower angles of the lumbar lordosis were decreased, but in the case of the backpack with reduced moment arm, the upper part of the lumbar lordosis was decreased, in the same way as with the general backpack; however, in the lower part, it was increased again. Previous studies showed that when the upper lumbar lordosis was decreased, the intervertebral disc of the lower lumbar was overextended and the whole lumbar lordosis was recovered to a certain degree [25]. When wearing a backpack with a reduced moment arm, the spine seems to have increased lumbar lordosis as a normal recovery mechanism. If the intervertebral disc space is narrowed due to problems such as aging, it is no longer possible to create an overextension state, resulting in a sagittal imbalance [25]. The backpack load can similarly compress the spinal discs, especially the lower lumbar discs [11]. Higher backpack loads resulted in lower disc height due to compression, making it difficult to create an overextension. This mechanism seemed to have contributed in part to the reduction of the lower lumbar spine in normal backpack wearing. However, in the backpack with reduced moment arm, the torque generated in the rear was reduced compared to the general backpack, which reduced the compression element of the disc, causing the lower lumbar overextension and recovering lumbar lordosis to a certain degree.

In this study, pelvic incidence was measured together, which is closely related to lumbar lordosis and does not change after growth. Characteristics of pelvic incidence do not change after growth, which is useful for determining the state of lumbar lordosis. In adult spinal deformity, the recovery of lumbar lordosis is important, and pelvic incidence is the criterion for determining how much to restore lumbar lordosis. Patients with large pelvic incidence should have more lumbar lordosis correction because of greater lumbar lordosis. Lumbar lordosis is known to be suitable for ± 9 degrees of pelvic incidence. Health-related disability was severe when the difference between pelvic incidence and lumbar lordosis was above 11 degrees [15]. In this study, after wearing a backpack, lumbar lordosis was decreased, which increased the difference between pelvic incidence and lumbar lordosis by more than 11 degrees. Long continuous wearing of the backpack may affect lumbar lordosis and cause imbalance in spinal sagittal alignment. In addition, the difference in pelvic incidence and lumbar lordosis was reduced when wearing a bag with reduced moment arm, and the level was restored to the level of no backpack. These data indicated that a bag with reduced moment arms is a design that can positively affect spinal sagittal alignment.

Sagittal balance is determined by the C7 plumb line [18]. The normal range of the C7 plumb line was defined as within 5 cm of the anterior and 2 cm of the posterior corner of the sacrum. Passing in front of 5 cm is called a positive sagittal imbalance, and 2 cm behind the back of the sacral spine is called negative sagittal imbalance. After wearing the backpack, the distance to forward increased significantly. In backpacks with reduced moment arms, the distance was reduced again, but this was not a statistically significant result. As lumbar lordosis was decreased, it moved forward of the spinal sagittal vertical axis. In this study, it seemed that lumbar lordosis was decreased and the anterior distance was increased. In addition, such changes are associated with the development of back pain and degenerative changes, which can be said to have adverse effects on spine health. Backpacks with reduced moment arms, although not statistically significant, showed a diminishing pattern and it might require multiple studies to increase this effect [26].

In previous studies, wearing a backpack tended to increase the head-on-neck extension (HNL), corresponding to the upper cervical spine angle, which reduces the LBL to move the load behind the back into the BOS. At the same time, it is reported that tilting the torso forward increased HNL to keep the gaze straight forward [9]. Upper and lower cervical lordosis had negative correlations [27]. The results of previous studies showed that the upper cervical spine angle tended to increase while wearing a backpack, while the lower cervical spine angle tended to decrease. In this study, we measured the angle of C2–C7, lower cervical lordosis, which is one of the most frequently used methods to analyze cervical spine alignment. As mentioned earlier, this angle was decreased after wearing a backpack. This position is also associated with the forward head position, which causes dysfunction of the musculoskeletal system of the neck and shoulders [17,28,29]. Recently, as the Internet penetration rate increases, people spend a lot of time in front of computers, which causes a recent issue, forward head posture [1]. It is known that as the forward head position is increased, lower cervical lordosis is decreased [17,30]. If the forward head position is continued, severe neck pain occurs due to abnormal strain on the capsular ligament due to excessive pressure on the facet joint, shortening of the posterior structure of the neck, and lengthening of the anterior structure [17]. The results of this study suggest that wearing a backpack may contribute to abnormal cervical spine alignment caused by recent life patterns. When wearing a backpack with a reduced moment arm, it was expected that the stress on the spine caused by the backpack would also be reduced, and the posture of the neck would be straightened as the change of the waist angle was reduced. However, the results of this study showed that the effect of wearing a reduced moment arm backpack on cervical spine alignment was not significant compared to when wearing a normal backpack. If the center of gravity of the backpack is located close to the axis of the spine, it has a significant effect on the torque change of the lumbar region in direct contact with the backpack, but the effect on the torque change of the cervical spine is negligible. However, in order to determine the exact mechanism, biomechanical studies on the changes in the torque of the cervical and lumbar spine caused by the backpack load will be needed.

Backpacks are the most common means of carrying in everyday life, so it is important to study not only standing posture but also walking posture [8]. Therefore, further discussion of spinal alignment will be needed during walking. In addition, lumbar lordosis was decreased in the results of this study, but the effect on other spinal alignment was small. Further multi-faceted studies analyzing spinal alignment and other variables, such as muscle activity, may be necessary to accurately understand the effects of backpacks on the body.

## 5. Conclusions

The results in this study showed that wearing a backpack caused an imbalance of sagittal spinal alignment, similar to the results of a previous study. In particular, it reduced lumbar lordosis, which is important for sagittal alignment and affected pelvic and cervical spine alignment. The backpack’s load caused stress in the back of the spine. The backpack designed in this study reduced the moment arm of the vertebral joint and separation. It was also shown to reduce lumbar alignment changes compared to conventional backpacks. Therefore, not only the weight of the backpack but also the center of gravity is a very important factor regarding spinal load. Further studies related to the design of backpacks are necessary for spine health.

## Figures and Tables

**Table 1:** Comparison of cervical spine alignment in each condition.

Parameter	WB	NB	RB	F	*p*
Cervical (degree)	11.4 ± 7.9	6.6 ± 4.5 *	6.5 ± 5.2 *	4.649	0.016

Values are expressed as mean ± standard deviation. WB, without backpack; NB, wearing normal backpack condition; RB, wearing backpack designed to reduce the load moment; * indicates a significant difference compared with WB.

**Table 2:** Comparison of lumbar spine alignment in each condition.

Parameter	WB	NB	RB	F	*p*
Whole arc (degree)	49.0 ± 6.6 ^#^	44.0 ± 8.3 *^,†^	47.6 ± 9.8 ^#^	5.583	0.008
Upper arc (degree)	9.3 ± 4.6	8.6 ± 3.4	8.5 ± 3.9	0.442	0.646
Lower arc (degree)	39.7 ± 3.2 ^#^	35.3 ± 5.8 *^,†^	39.1 ± 7.8 ^#^	6.783	0.003

Values are expressed as mean ± standard deviation. WB, without backpack; NB, wearing normal backpack condition; RB, wearing backpack designed to reduce the load moment; * indicates a significant difference compared with WB, ^#^ indicates a significant difference compared with NB, ^†^ indicates a significant difference compared with RB.

**Table 3:** Comparison of differences between pelvic incidence and lumbar lordosis in each condition.

Parameter	WB	NB	RB	F	p
Differences (degree)	10.0 ± 9.4 ^#^	13.7 ± 9.0 *	12.0 ± 9.2	3.307	0.049

Values are expressed as mean ± standard deviation. WB, without backpack; NB, wearing normal backpack condition; RB, wearing backpack designed to reduce the load moment; * indicates a significant difference compared with WB, ^#^ indicates a significant difference compared with NB.

**Table 4:** Comparison of sagittal vertical axis in each condition.

Parameter	WB	NB	RB	F	p
SVA (mm)	25.2 ± 16.8 ^#^	49.3 ± 16.1 *^,†^	46.9 ± 11.2 ^#^	10.837	0.001

Values are expressed as mean ± standard deviation. SVA, sagittal vertical axis; WB, without backpack; NB, wearing normal backpack condition; RB, wearing backpack designed to reduce the load moment; * indicates a significant difference compared with WB, ^#^ indicates a significant difference compared with NB, ^†^ indicates a significant difference compared with RB.
